# Conservative versus surgical management of mandibular condylar fractures: a comparative study

**DOI:** 10.2340/aos.v85.46063

**Published:** 2026-06-15

**Authors:** Hampus Eksell, Anna Jinghede Sundwall, Robin Radamson, Annika Rosén, Robert Heymann

**Affiliations:** a General Dental Practitioner | Partner at WE Dental AB, Nacka, Sweden; b Forensic Section, The Swedish Police Authority, Police Region Bergslagen, Örebro, Sweden; c Department of Oncology and Pathology, Karolinska Institutet, Solna, Sweden; d Public Dental Services, Örebro, Sweden; e Department of Oral and Maxillofacial Surgery, Eastman Institute, Stockholm, Sweden; f Department of Clinical Dentistry, University of Bergen, Bergen, Norway; g Department of Oral and Maxillofacial Surgery, Karolinska University Hospital, Stockholm, Sweden; h Department of Dental Medicine, Karolinska Institutet, Stockholm, Sweden

**Keywords:** Mandibular condyle fracture, open reduction internal fixation, closed reduction, intermaxillary fixation, treatment outcome, complications

## Abstract

Condylar process fractures are a common mandibular injury predominantly affecting males, with a male-to-female ratio of 3:1. This study examines outcomes in patients with mandibular condylar fractures treated either conservatively or surgically, comparing long-term subjective results. A total of 281 cases were reviewed (144 treated conservatively and 137 treated surgically), with symptoms and functional outcomes assessed through questionnaires.

Surgical intervention was more common in complex cases (e.g. bilateral fractures or concurrent maxillofacial injuries), and the surgical treatment group had a higher proportion of male patients and trauma related to violence. Mandibular joint sounds (e.g. clicking and crepitation) were more frequent in patients treated conservatively, whereas pain both at rest and during movement was more common in women and those with interpersonal violence-related trauma. Multivariable analysis indicated the presence of concurrent fractures as a risk factor for joint sounds, and gender and trauma type influenced pain and symptom frequency.

Despite these differences, both treatments achieved comparable success in maintaining stable occlusion and achieving painless jaw function. These findings suggest that both conservative and surgical treatments can be effective, although individual factors such as gender, trauma type and concurrent fractures may affect post-treatment symptomatology and choice of treatment.

## Introduction

Condylar process fractures of the mandible are common in facial trauma patients, with an incidence ranging from 18 to 52% of all mandibular fractures [1, 2]. Research has shown variations in the causes of mandibular fractures across countries and age groups, with road traffic accidents (RTA), falls and alleged assaults being the main contributors [1–3]. Males appear to sustain condylar fractures substantially more often than females, with a reported male-to-female ratio of 3:1 [1, 3].

The mechanism of condylar fractures can be either direct or indirect, and outcomes depend on factors such as the direction, degree and energy of impact, as well as the patient’s dental and occlusal status [2]. Treatment options include conservative approaches, with or without intermaxillary fixation (IMF), or surgical intervention through open or endoscopic reduction and internal fixation. The goal of treatment is to restore stable occlusion, full mandibular function and prevent long-term dysfunction or pain [4, 5]. However, the choice between conservative or surgical treatment remains debated [5–7]. Conservative treatment minimises risks associated with surgery, such as infections, nerve damage and scarring [8, 9]. However, over the past two decades, surgical approaches have become increasingly favoured – particularly in cases involving condylar displacement, vertical height loss or concomitant facial fractures – due to their improved ability to restore the temporomandibular joint (TMJ) and mandible to their correct anatomical form and position [10–15]. Surgery may reduce the risk of post-treatment complications such as malocclusion, facial asymmetry, chronic pain and TMJ mobility issues [7, 11, 14]. Regardless of treatment modality, outcomes are influenced by factors such as fracture location, displacement severity, patient age and health and coexisting fractures [16, 17].

Despite an ongoing debate over optimal treatment, few long-term studies have compared outcomes of conservative versus surgical management [5, 6, 18]. Therefore, this study aimed to retrospectively evaluate and compare subjective outcomes between these two treatment approaches.

## Materials and methods

This was a retrospective follow-up study on treatment outcomes in patients with mandibular condylar fractures, which involved two parts: data collection from medical records (Part I) and patient self-reported symptoms via a questionnaire, together with a letter of consent (Part II), in conservatively treated patients (CTP) and surgically treated patients (STP). Ethical approval was granted by the Regional Ethical Review Board in Stockholm, Sweden (no 2015-62-31, no 04-1024/3), prior to the commencement of the study. The study adhered to the guidelines of the World Medical Association, the Declaration of Helsinki and Good Clinical Practice [19].

Patients with mandibular condylar (both extra- and intra-capsular) fractures treated conservatively (CTP) between 1994 and 2005 and surgically (STP) between 2005 and 2013 at the Department of Oral and Maxillofacial Surgery, Karolinska Institutet, Huddinge and Karolinska University Hospital, Solna, were included in this study. The study was based on data from two separate research projects, resulting in two groups from different time periods. During each period, both treatment techniques were used but the choice at that time was to only collect data from one treatment in each period. Patient selection was based on diagnostic codes and records from the Patient 2000 and Take Care journal systems. No exclusion criteria were applied for Part I. Part II excluded only edentulous patients. Both study parts included patients with concurrent maxillofacial fractures.

Data were retrieved by means of a standardised protocol from patient records for each patient, including personal information (gender assigned at birth based on Swedish personal identity number, year of birth and address), age at the time of trauma, cause of trauma, date of trauma (year and month), type of fracture(s) (uni-/bilateral, right and/or left), concurrent fractures and type of treatment (soft diet and/or IMF or surgical treatment).

Subjective post-treatment outcomes were assessed via a questionnaire comprising dichotomous (‘yes’ or ‘no’) and multiple-choice questions. All patients received the questionnaire, a consent form and an information letter about the study (see Appendix C). The CTP questionnaire differed slightly from the STP version to accommodate STP and included additional questions on subjective experiences (see Appendices A and B). Data from both groups were compiled in Microsoft Excel, where each patient was assigned a unique identification number.

### Statistical analysis

Descriptive statistics included mean values, ranges and standard deviations. Group comparisons utilised Fisher’s exact test for binary variables, the chi-squared test for nominal categorical variables and Fisher’s permutation test for numerical variables. All analyses were two-sided with statistical significance set at *p* < 0.05. Multivariable analyses were conducted using logistic regression, with binary outcomes reversed for easier interpretation of odds ratios (ORs), as dependent variables (e.g. ‘not fully recovered’, ‘cannot bite off large pieces’ and ‘pain at rest’) and independent variables (e.g. age at trauma, treatment type, gender, trauma cause and concurrent fractures). Additional analyses used linear regression with the number of subjective discomforts as a continuous outcome variable. Associations were reported as ORs with 95% confidence intervals (CIs).

## Results

A total of 144 CTP and 137 STP cases were included in the study, part I. Data analysis showed that 52 CTP patients (36.1%) were treated with IMF, another 52 (36.1%) with a soft diet and treatment details were unavailable for the remaining 40 cases (27.8%). Males predominated in both groups, comprising 61.1% (*n* = 88) of the CTP group and 77.2% (*n* = 105) of the STP group, with a significantly higher proportion of men in the STP group. The mean age was 40.8 years (range = 4–92 years) for the CTP group and 38.8 years (range = 15–89 years) for the STP group. Falls (44.0%) were the most common cause of injury in the CTP group, whereas violence (34.6%) was predominant among the STP group. Bilateral fractures occurred twice as frequently in the STP group compared to the CTP group (26.7% vs. 13.3%, respectively), and concurrent fractures were over three times more common in the STP group (64.2% vs. 17.4%, respectively). Mandibular corpus and angulus fractures were the most prevalent among all cases (Table 1).

**Table 1:** Demographic data, cause and type of injury from patients’ records

	CTP *n* = 144	STP *n* = 137	CTP vs. STP *p*-value**
Age at trauma (mean ± SD)	40.8 ± 21.1	38.8 ± 17.3	> 0.300
Gender (% women)	38.9	22.8 (*n* = 136*)	**0.005**
Cause of trauma:	*n* = 134*	*n* = 130*	
Violence (%)	23.1	34.6	0.054
Fall (%)	44.0	29.2	**0.018**
Bicycle (%)	17.2%	13.1	> 0.300
Type of fracture:	*n* = 134*	*n* = 135*	
Bilateral (%)	13.3%	26.7	**0.008**
Concurrent fracture (%)	26.4	74.5	**< 0.001**
Corpus/angulus (%)	17.4	64.2	**< 0.001**
Ramus/condyle (%)	0.0	3.7	0.053
Maxilla/midface (%)	10.4	22.6	**0.009**
Other (%)	0.0	2.9	0.110
Unknown (%)	0.0	1.5	> 0.300

CTP: conservatively treated patients; STP: surgically treated patients; SD: standard deviation.

* The number represents patients with available data.

** *p* < 0.05 marked with bold text.

A total of 77 CTP and 64 STP completed the questionnaire in part II, yielding a response rate of 50.2%. Gender distribution among CTP respondents was nearly equal (48.1% women), whereas men predominated among the STP respondents (67.2% men). The mean age was 38.0 years (range = 5–92 years) for the CTP group and 42.1 years (range = 15–78 years) for the STP group. Falls were the most frequent cause of injury in the CTP group, whereas interpersonal violence was the primary cause among STP patients.

Bilateral fractures were significantly more prevalent in the STP group compared to the CTP group (40.3% vs. 14.3%, respectively), and concurrent fractures were also significantly more frequent in the STP group (75.0% vs. 28.6%, respectively). The only subjective area of discomfort that was significantly different between groups was clicking and crepitation (Table 2). Both CTP and STP patients with concurrent fractures reported similar symptom rates (73%, *n* = 16 for CTP; 79%, *n* = 38 for STP).

**Table 2:** Data from patients who answered the questionnaire, part II.

	CTP *n* = 77	STP *n* = 64	CTP vs. STP *p*-value**
Age at trauma (mean ± SD)	38.0 ± 20.9	42.1 ± 16.9	0.250
Gender (% women)	48.1	32.8	0.096
Cause of trauma:	*n* = 72*	*n* = 61*	
Violence (%)	16.7	21.3	> 0.300
Fall (%)	45.8	32.8	0.180
Bicycle (%)	18.1	23.0	> 0.300
Type of fracture:	*n* = 77	*n* = 62*	
Bilateral (%)	14.3	40.3	**< 0.001**
Concurrent fracture (%)	28.6	75.0	**< 0.001**
Number of subjective discomforts (0–8) (mean ± SD)	2.1 ± 2.2	1.8 ± 1.9	> 0.300
Not fully recovered (%)	31.6	44.4	0.180
Cannot bite off large pieces of food (%)	15.8	9.5	> 0.300
Cannot bite off small pieces of food (%)	7.8	12.2	> 0.300
Not occlusion resemblance (%)	49.4	39.3	> 0.300
Pain at movement (%)	14.5	19.4	> 0.300
Pain at rest (%)	14.3	12.9	> 0.300
Hard to open mouth (%)	31.2	23.4	> 0.300
Clicking/crepitations (%)	41.6	21.9	**0.013**

CTP: conservatively treated patients; STP: surgically treated patients; SD: standard deviation.

* The number represents patients with available data.

** *p* < 0.05 marked with bold text.

**Figure 1:**
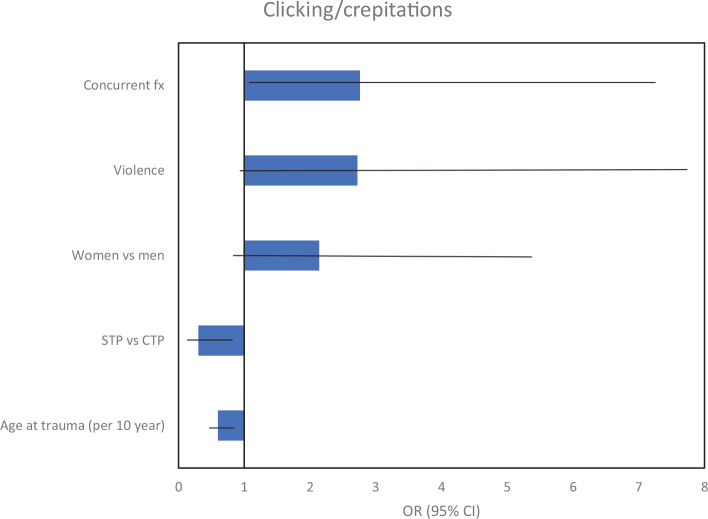
Multivariable associations between baseline variables (independent) and clicking/crepitation (dependent). Associations are described as odds ratios (ORs) and 95% confidence intervals (CIs).

Clicking/crepitation was more common among the CTP group, both as an univariable analysis (*p* = 0.013) (Table 2) and after adjustment for age, gender, violence as the cause of trauma and concurrent fracture (*p* < 0.001). In the multivariable analysis, older age was a protective factor for clicking/crepitation (*p* < 0.001), and concurrent fracture was identified as a risk factor (*p* = 0.039) (Figure 1).

Women had a higher risk of being unable to bite off large pieces of food (*p* = 0.015), experiencing pain at rest (*p* = 0.028) and pain during movement (*p* = 0.010), after adjustment for age, treatment type, violence as trauma cause and concurrent fractures. Additionally, women showed a trend towards subjectively incomplete recovery in this multivariable model (*p* = 0.056). Violence as a trauma cause was a risk factor for being ‘unable to bite off large pieces of food’ (*p* = 0.028), ‘experiencing pain during movement’ (*p* = 0.041) and ‘pain at rest’ (*p* = 0.0043) after adjustment for age, treatment, gender and concurrent fractures. Patients with violence-related trauma also showed trends towards changes in occlusion (*p* = 0.086) and clicking/crepitation (*p* = 0.061), and violence was more common among men (*p* < 0.001). Facial nerve damage was reported by 15 STP patients (22.4%). Both female gender and violence-related trauma were significant risk factors for a higher number of subjective discomforts (*p* = 0.014 and *p* = 0.037, respectively) after adjustment for treatment, age and concurrent fractures. A trend was also observed towards more numbers of subjective discomforts in patients with concurrent fractures (*p* = 0.084). No symptom differences were found between CTP treated with a soft diet and those with IMF despite a higher prevalence of concurrent fractures in the latter group (42.3% vs. 13.8%, respectively).

## Discussion

In this study, mandibular condylar fractures were observed to be over twice as common among men as women, aligning with previous findings [1, 2, 20]. Men also predominated in surgical treatments, with 49% (*n* = 140) of all patients presenting with concurrent fractures, of whom 74.3% (*n* = 104) were men. Bilateral fractures occurred in 19% (*n* = 54) of patients. These findings are consistent with earlier research indicating that patients exposed to facial trauma often sustain multiple maxillofacial fractures [1, 2]. Additionally, the STP group had significantly higher incidences of both concurrent and bilateral fractures compared to the CTP group, suggesting a preference for surgical treatment in cases of greater fracture complexity [1, 12]. Several studies have indicated that surgical intervention better facilitates optimal anatomical positioning, restoration of ramus height, stable occlusion and improved subjective post-treatment outcomes [9, 12, 14, 21].

Our findings also suggest that men are more frequently affected by condylar, concurrent and bilateral fractures and are more likely to undergo surgical intervention. This gender imbalance may be attributed to men’s higher involvement in high-risk activities associated with complex maxillofacial injuries, such as interpersonal violence and RTA. In contrast, women are more commonly affected by condylar fractures from falls (60.7%) and bicycle accidents (17.9%) [2, 22].

Regardless of treatment method, condylar fractures are associated with various post-treatment symptoms [1, 9, 18]. In our cohort, the only significant difference between the groups was the higher frequency of joint sounds, 41.6% of patients in the CTP group compared with 21.9% in the STP group, corroborating findings by Tabrizi et al. [23]. Our multivariable analysis also indicated that concurrent fractures in both groups were a risk factor for post-treatment clicking and crepitation which aligns with Tabrizi et al. [23]. However, clicking and crepitation are multifactorial in origin and may be related to trauma-induced TMJ misalignment, teeth grinding, osteoarthritis or disc derangement, with a reported prevalence of 13–35% [24, 25].

Following mandibular trauma, post-treatment joint sounds are associated with condylar disc derangement, ligament elongation, disc deformation and displacement [25]. Condylar fractures can also alter the condyle’s position within the temporal fossa, leading to TMJ dysfunction and subjective discomfort [26, 27]. Helmer LML et al argued that restoring the ramus height and condylar anatomy is crucial for functional TMJ recovery [28]. Therefore, it may be inferred that certain pre-treatment conditions, such as height loss and condyle dislocation, could benefit from surgical intervention [29]. Future studies should focus on the CTP subgroup with persistent joint sounds to identify specific indications for surgical treatment.

In this study, post-treatment pain, as subjective outcome, was significantly associated with female gender and trauma caused by interpersonal violence. Such factors were also linked to a higher frequency of reported symptoms, aligning with the broader research on intimate partner violence, which disproportionately affects women and may contribute to various health issues such as chronic pain and temporomandibular disorder (TMD) [30]. Psychological stress from trauma can also induce bruxism, exacerbating TMD and influencing subjective outcomes after condylar trauma treatment [30]. In the current study, patients with concurrent fractures reported slightly higher symptom scores, potentially due to the involvement of additional facial structures. However, no difference in the number of symptoms was observed between CTP and STP with concurrent fractures, suggesting that even complex fractures can be effectively managed conservatively, leading to satisfactory outcomes. These findings (no difference in the number of symptoms) are not consistent with Bera RN et al., who reported that severe and complex fractures with condylar displacement are better treated with a surgical approach [15]. Shobha ES et al. reported that the outcome regarding mouth opening, laterotrusion and protrusion favours the CTP [31]. One can conclude that the treatment of condylar process fracture is an ongoing matter of controversy.

A satisfactory functional outcome after condylar fracture treatment typically involves adequate mouth opening, stable occlusion, painless jaw movements and minimal morbidity [5, 20]. Recent meta-analyses indicate that there are overall no significant differences between conservative treatment and open reduction and internal fixation (ORIF), except that ORIF provides less deviation [15]. Improving patient education on expected outcomes could further enhance satisfaction by aligning treatment results with patient expectations [32].

### Limitations

This study has certain limitations, the foremost of which is the small sample size. This was a retrospective study, and the data were derived from two primary sources: patient records and self-reported data. However, not all medical records included complete information on each parameter, which is a common limitation of retrospective studies. Additionally, self-reported data are subject to recall bias, potentially affecting accuracy. Finally, data from the two groups were collected at different times. It was previously more common with conservative treatment (even in severe trauma). However, surgical techniques have since then been refined (regarding fixation device, minimal invasive technique, improved intraoperative guidance and better understanding of fracture biology) and are now more commonly used in fracture management (especially on patients with severe trauma). Future studies, such as randomised controlled trials, are warranted to address these limitations.

## Conclusion

To conclude, patients with concurrent fractures are more likely to receive surgical treatment. Apart from a higher incidence of clicking and crepitation in conservatively treated fractures, there is no significant difference between surgical and conservative treatment in terms of patient-reported function and pain.

Female patients and those with trauma due to interpersonal violence are at greater risk of reporting pain and experiencing more subjective discomforts.

Given the limited differences in patient-reported outcomes between treatment approaches, the findings indicate that both conservative and surgical approaches result in comparable long-term subjective outcomes in terms of function and pain. Conservative treatment appears to be a reasonable first-line option due to its less invasive nature. However, surgical treatment appears to be more commonly utilised in cases with greater fracture complexity.
